# Phage Therapy Faces Evolutionary Challenges

**DOI:** 10.3390/v10060323

**Published:** 2018-06-12

**Authors:** Clara Torres-Barceló

**Affiliations:** University of Reunion Island, UMR Plant populations and bio-agressors in tropical environment (PVBMT), Saint-Pierre 97410, Reunion, France; clara.torres@cirad.fr; Tel.: +262-262492727

**Keywords:** phage therapy, evolution, bacterial resistance, virulence

## Abstract

Antibiotic resistance evolution in bacteria indicates that one of the challenges faced by phage therapy is that, sooner or later, bacteria will evolve resistance to phages. Evidently, this is the case of every known antimicrobial therapy, but here this is also part of a ubiquitous natural process of co-evolution between phages and bacteria. Fundamental evolutionary studies hold some clues that are crucial to limit the problematic process of bacterial resistance during phage applications. First, I discuss here the importance of defining evolutionary and ecological factors influencing bacterial resistance and phage counter-defense mechanisms. Then, I comment on the interest of determining the co-evolutionary dynamics between phages and bacteria that may allow for selecting the conditions that will increase the probability of therapeutic success. I go on to suggest the varied strategies that may ensure the long-term success of phage therapy, including analysis of internal phage parameters and personalized treatments. In practical terms, these types of approaches will define evolutionary criteria regarding how to develop, and when to apply, therapeutic phage cocktails. Integrating this perspective in antimicrobial treatments, such as phage therapy, is among the necessary steps to expand its use in the near future, and to ensure its durability and success.

## 1. Introduction

Phage therapy is gradually becoming a reality in clinical, veterinary, and agricultural settings [1,2,3]. In order to avoid the past mistakes of chemical treatments, it is important to prevent bacterial resistance to phages where possible. A review and an expert comment in this same Special Issue advocate for a molecular and evolutionary combined basis in the selection of therapeutic phages [4,5]. Phage cocktails, in particular, are given special attention, and represent an excellent method to face bacterial genetic variability and prevent the evolution of resistance. In the past, a large host range was the main criterion to select different phages [6,7]. The newly published review suggests an expansion in phage choice to specifically include phages that target different bacterial receptors and phages with counter-defense abilities. Also considered are the phages’ resistance to environmental factors (e.g., pH and temperature) and phages’ viability, which could facilitate storage and production. In the expert opinion piece, the researchers suggested the use of pre-adapted, or “trained”, phages to overcome the resistance capacity of bacteria, a method that has proven to be successful in the past. Both articles point out that continued exposure to phages may activate the immune system, increasing their elimination within the body. The experts bring their attention to the fact that overuse of phages could result in the evolutionary selection of resistance to phages in bacteria, which, in a similar light to antimicrobials, could be transferred to clinical situations via horizontal gene transfer. To avoid this, the authors consider the use of personalized medicine as a means to develop a successful and sustainable phage therapy strategy. Here, I present additional evolutionary concerns that can guide the design of therapeutic treatments with phages. Some concepts are open questions still in need of confirmation in the laboratory, clinic, or in vivo settings. Altogether, these are perspectives that may increase the benefits of any antimicrobial strategy.

## 2. Factors to Consider Regarding the Different Mechanisms of Resistance to Phages and Counter-Defense

The interaction between phages and bacteria stands as one of the fastest and more assorted evolutionary processes on Earth. Not only do bacteria and their parasites have short generation times, large population sizes, and high mutation rates, there are also bacteria and phages of all kinds and in all sorts of environments [8]. This co-evolution likely centers around resistance in bacteria and counter-resistance of phages. Among the resistance mechanisms active in bacteria facing phages are phenotypic shifts [9], point mutations in surface structures used by phages as receptors [10], or acquisition of CRISPR (Clustered Regularly Interspaced Short Palindromic Repeats) spacers [11]. Additionally, new and diverse bacterial defense mechanisms have been described lately [12]. Phages in turn can avoid bacterial defenses by, for instance, modifying their life cycle parameters (burst size, lysis time, etc.) [13], mutating receptor binding proteins [10], or recombining with other viruses [14].

**Figure 1:**
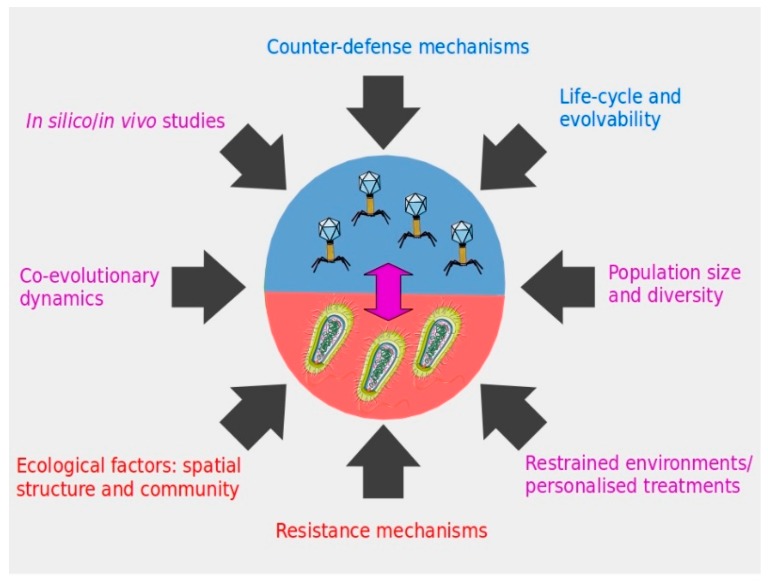
Different factors to consider in phage therapy applications in order to ensure the durability of the strategy against pathogenic bacteria. Color code associates the factors to therapeutic phages (blue), the targeted bacteria (red), or both microorganisms and the interaction between them (violet).

Determining which resistance or counter-defense processes will take place at any given moment will depend on several factors, the understanding of which can help to predict the outcome of phage–bacteria interactions (Figure 1). First, there is the probability of occurrence of any resistance and counter-resistance mechanism, conditioned by mutation rate, and phage and bacterial population diversity. Besides internal microbial factors, parameters such as nutrient availability will determine the growth rate of bacteria (and their obligatory parasites), and thus the frequency of resistance generation. Second, there is the ecology of the infection environment, including the spatial structure. This is illustrated in the mammalian gut, where phages adhere to the mucus and attack invasive bacteria [15], whereas other body sites are less prone to phage–bacteria interaction [16]. Furthermore, the microbial communities present could both facilitate or impede phage therapeutic action. For example, their competitive interaction with the pathogenic strain will impose an additional fitness cost, which could enhance phage control, whereas a complex community can complicate the chance phages have to encounter their specific target. Third, a decisive parameter is the fitness cost imposed by each defense and counter-defense mechanism on phages and bacteria. For example, it has recently been shown that the CRISPR-Cas system as a resistance mechanism in *Pseudomonas aeruginosa* is more likely to be selected or maintained if the same host frequently faces the same phage [17]. In this example, the authors demonstrated that the absolute cost of receptor modification was lower than the inducible cost of adaptive immunity (provided by a CRISPR-Cas system), unless facing a static target. Thus, the presence of a phage defense mechanism (e.g., CRISPR-Cas) in a bacterium targeted for therapy does not always mean that it will be crucial for resistance to a therapeutic phage, especially if it is part of a diverse cocktail.

On the phage side, their small genome size may restrain their evolutionary capacities, favoring the selection of non-pleiotropic and less costly mutations. For example, modifications on genes coding for receptor binding proteins have been more frequently observed when compared to mutations in the phage polymerase, a core enzymatic activity [10,11]. Interestingly, a recent study proves that at least some phages are able to extend their genome size while exposed to bacteria in natural environments [11]. For therapeutic purposes, a bigger genome size and fewer overlapping genes in phage candidates may indicate potential for evolution and the capacity to overcome bacteria, although this idea remains to be tested. In conclusion, when exploring resistance/infectivity mechanisms in these microbes, one needs to consider the evolutionary forces favoring their selection (Figure 1). Also, the life-history of the pathogen and its past encounters with phages may determine the success of any therapeutic phages applied. Genomic tools are unveiling new and fascinating defense mechanisms, but without understanding their biological relevance, this knowledge is just a hint of its essence.

## 3. Co-Evolutionary Dynamics between Phages and Bacteria Influence the Therapeutic Outcome

Co-evolutionary dynamics between phages and bacteria can differ relative to time and genetic variability in the population of microbes, influencing the probability of resistance. Deeper understanding of these processes could point to the conditions that can ensure a successful treatment against pathogenic bacteria (Figure 1). It has been shown experimentally that an arms-race dynamics (ARD) type of evolutionary process can change to fluctuating selection dynamics (FSD), depending on the phase of the interaction [18]. This was observed in *P. fluorescens* and its phage phi2, a well-studied model of co-evolution, over a hundreds of generations (60 transfers) experiment. In the first stages of a bacteria-phage encounter (first 10 transfers), more types of resistance and infectivity alleles were available, which imposed lower fitness costs than in later transfers. Afterwards (between transfers 10 and 50), alleles of defense and attack were more limited, and their selection fluctuated depending on the most abundant genotypes of phages and bacteria in the environment, and likely imposed a higher fitness cost to both microbes. Co-evolutionary dynamics may be different for other phage-bacteria pairs but, in this particular case, it is plausible that an ARD type of interaction between the targeted bacteria and a “trained” therapeutic phage will be a very efficient strategy. In an FSD process, the probability of success is likely to be lower as the resistance/infectivity process is strongly frequency dependent. Conversely, the higher fitness costs confronted by bacteria during FSD may benefit therapeutic phages in the evolutionary race.

To recapitulate, in studying the evolution of phage resistance in bacteria, short- and long-term scales must be considered. Experimental evolution approaches encourage researchers to consider bacteria–phage interactions further than 24 h to account for the evolvability of both microorganisms [19]. Different effects have been observed experimentally when looking at short- or long-term co-evolving phages and bacteria. As stated before, evolutionary dynamics may differ over time, e.g., early arms-race *versus* late fluctuating dynamics [18]. It has also been shown that in combination therapies using phages and antibiotics, the control of pathogenic bacteria was more efficient in the first stages of the exposure (e.g., [20]). In practical terms, quantifying the capacity of the resistance of each strain, and performing co-evolutionary studies with candidate phages, could guide and improve phage therapy. Studies such as those just mentioned can direct efforts towards the characterization of the situations (e.g., timing, type of bacterial population, etc.) most likely to be controlled by phages.

Similarly, the genetic variability and population size (i.e., standing genetic variation) of the targeted bacteria will probably increase the diversity of the type of evolutionary dynamics and defense mechanisms selected [17]. In the course of a phage therapy treatment, input of new bacteria or conditions that favor a high microbial growth rate (e.g., immunocompromised patients, resource availability, etc.) may contribute to a higher probability of developing resistance to phages. In those conditions, a more varied phage cocktail and/or higher initial inoculum (MOI: multiplicity-of-infection) may be advised as a therapeutic strategy. Carrying on with the rationale of the expert comment published in this journal, in intensive veterinary or agricultural settings, the large population size of bacteria and the number of phages required for treatments will enhance the likelihood of resistance [5]. Notwithstanding, the extensive spread of phage resistance in bacteria (e.g., via horizontal gene transfer) in this type of natural framework remains to be decidedly proven. Smaller agricultural or veterinary frameworks and contained environments (e.g., plant nurseries, tool disinfection, etc.) are more adapted and likely to produce a successful outcome of the use of therapeutic phages. This is the reasoning behind, for example, the Listex and Biolyse preventive phage preparations against *Salmonella* and *Pectobacterium* pathogens currently used in packed products in the food industry [3,21]. Timely use of phages as a preventive treatment will target a smaller population of bacteria, since it is a more effective and less risky scenario. In conclusion, the epidemiology and ecology of bacteria and phages must certainly be integrated into disease management before any therapeutic incursion against rapidly evolving microbes (Figure 1).

## 4. Managing Disease: Towards Sustainability

Current knowledge suggests that the way antimicrobial treatments influence virulence parameters in bacteria are important to controlling infectious diseases in the longer term [22]. Increasingly, medical approaches include the evolutionary perspective, where the aim is not to eliminate pathogens completely, but to reduce or impair their population. This implies to apply a weaken selection pressure on the pathogens, or to target and dismantle virulence-specific mechanisms. In order to restrain disease, this approach often relies on the role of the host’s immune system or the local microbiota.

In phage therapy, it is conceivable to alter or contain the virulence, and phage or antibiotic resistance emergence of bacterial pathogens. When selecting a therapeutic phage, the frequency of bacterial resistance induced by every particular phage is an advisable parameter to be examined. Several factors should be explored related to the facilitation of phage resistance in bacteria or the ability of phages to counteract it. Together, the phage genome size, mutation rate, and burst size could all be features determining the speed and potential adaptation of phages to bacteria. A large burst size increases the probability that phages contact target bacteria, the first step of infection. The amplification ability of phages could compensate the advantage of antibiotics at better diffusing in the body, and therefore reaching the site of infection. If phages can eliminate bacteria faster than they can replicate, a high burst size also results in a lower risk of selection for phage-resistant bacteria. At the same time, however, these phages imply a stronger selective pressure for bacteria, and could lead to faster or stronger resistance. An open question would then be trying to understand whether there is an evolutionary optimum of phage virulence that could constrain bacterial resistance evolution over longer periods of time.

The type of receptors desired for therapeutic phages is one of the most important parameters, and is frequently discussed (e.g., [4]). Are receptors such as lypopolysaccharides (LPS) more likely to be mutated (i.e., resistant) because of their intrinsic variability? Or are porins and pili structures easier to be modified, or their expression repressed in bacteria? It will likely depend on their environment and the fitness impact each mutation accrues. Interestingly, both LPS and pili are virulence factors that can be modified via selection, decreasing virulence in phage resistant bacteria. Several examples in humans, plants, and animals have demonstrated the role of phages in decreasing bacterial virulence [23,24,25]. Experimental evolution approaches may help elucidate which receptors favor phage treatment in the long term, i.e., enhance phages’ adaptation. This is the case in a study from Betts and collaborators, where they find that *P. aeruginosa* phages targeting LPS receptors show ARD compared to phages targeting pili, which undertake FSD patterns [26]. Their interpretation is that pili are retractable structures, whereas the LPS is an essential component of the bacteria membrane whose complexity allows for ARD sequential changes. Consequently, phages attaching to LPS receptors may be interesting for their wide and efficient potential of evolution. In contrast, loss of pili is associated with a high fitness cost in bacteria depending on the environmental conditions and the bacteria are only temporarily modified. From a therapeutic perspective, conclusions are mixed but worth further exploration.

A much clearer case are phages targeting mechanisms associated with antibiotic resistance in bacteria. Although these phages can be engineered [27], they are also found in nature. The phage PRD1 of *Escherichia coli* uses proteins encoded by plasmids as receptors, selecting against bacteria that contain conjugative plasmids, which are mobile structures that carry and spread antibiotic resistance genes [28]. Another example of phages selecting against antibiotic resistant bacteria is that of phage OMKO1 of *P. aeruginosa.* This phage recognizes a cell surface protein that is part of the multi-drug efflux system as the bacterial receptor. Phage-resistant bacteria harbor mutated efflux pumps that are ineffective for antibiotic resistance [29]. At least one case of compassionate use of this last phage has been successful [30], prompting further treatments.

Different researchers and clinicians have advocated for applying phage therapy as a personalized medicine for different reasons, including a reduced selection for phage resistance in bacterial populations. First, a moderate (personalized versus generalized) use of phages could avoid strong evolutionary pressures on bacterial populations derived from high doses found with other antimicrobials [31,32]. Second, as stated in the recent articles of this Special Issue, sensitization of the immune system to phages and their elimination after repeated use should not be discarded. Third, a tailored phage strategy, such as the “magistral preparation” recently approved in Belgium [33], is low-priced and fast compared to the standard drug licensing pathway. The best way to ensure the durability of phage therapies’ efficacy is the careful application of phage therapy, minimizing past mistakes with other antimicrobials; personalized phage therapy currently seems a favorable procedure to accomplish this.

## 5. In Silico and In Vivo Studies are Necessary to Understand Bacteria–Phage Evolution

In community ecology and evolutionary studies, the interaction of bacteria and their phages has become a cornerstone field [34]. However, the majority of these are in vitro studies; while in the wild, with diverse microbial communities, abiotic factors or host colonization by bacteria, co-evolutionary dynamics appear much more complex (e.g., [35]). Indeed, bacterial phage resistance is a condition that potentially implies metabolic and evolutionary costs, which are not always accounted for in vitro analyses [36,37,38]. Different studies have proved that a phage candidate’s effect in vitro does not always relate to their capacity to control the disease in vivo (e.g., [39]). It has been suggested that different phage resistance mechanisms are selected in bacteria depending on the ecological conditions [17], although this remains to be largely explored in vivo. A recent study proved that phage evolution differed between dixenic mice and planktonic cultures [40]. Additionally, it has been demonstrated that a synergism between phages and the immune system is essential to wipe out pathogens from hosts [41]. Environmental complexity, the host immune system, differential bacterial gene expression, evolutionary trade-offs, or the interactions with diverse intra- and inter-microbial communities play a significant role in this regard. In order to understand evolutionary pressures, assays in both conditions are necessary.

The design and use of phage cocktails for therapy certainly allows for combating bacterial resistance evolution. The underlying idea is to deploy phages with complementary systems of attack for the same bacterium, while covering the entire (or as much as possible) target bacterial variability. Interactions between phages, either competition or facilitation, may exist in phage combinations (as in any ecological community), and make the efficiency of the cocktail deviate from the addition of the phage isolates’ effects [42]. In addition to purely experimental approaches, computer simulations and algorithms of the interaction networks between phages and bacterial populations could help to guide the design of phage cocktails, defining characteristics of phage assemblages that are key to optimizing the cocktail stability and efficiency [43]. Understanding these complex dynamics may therefore help in choosing phages that increase the efficiency of a cocktail and aid in determining the frequency at which the cocktail should be applied in real systems.

## 6. Conclusions

It is essential in the design of effective antimicrobial strategies to consider the evolution of resistance in bacteria. Our understanding of phage–bacteria interactions can guide the selection of specific combinations that can help to minimize any possible impact of resistance development in an effective therapy. Including diverse approaches using different pathogenic bacteria will set up evolutionary principles to refine the selection of candidate phages. In other words, we must aim to detect potentially long-term effective phages to be used as durable control strategies. Among the evolutionary criteria and unsolved questions related to phages and their effect in bacteria are the following: timing of application, effects of bacterial population diversity and life-history related to phages, as well as phage features regarding their potential of adaptation to bacteria. All this information will help compile data on the capacity of each phage to select resistant bacteria and aid in choosing effective phages accordingly. Future research should provide much-needed results on the evolutionary and molecular consequences of phage therapy treatments in complex environments. Evolutionary approaches can provide insights into how to limit the evolution of bacterial resistance to phages and truly advance phage therapy, a potential solution to several worldwide problems.

## References

[1] Kingwell K. (2015). Bacteriophage therapies re-enter clinical trials. Nat. Rev. Drug Discov..

[2] Buttimer C., McAuliffe O., Ross R.P., Hill C., O’Mahony J., Coffey A. (2017). Bacteriophages and Bacterial Plant Diseases. Front. Microbiol..

[3] Moye Z., Woolston J., Sulakvelidze A. (2018). Bacteriophage Applications for Food Production and Processing. Viruses.

[4] Casey E., van Sinderen D., Mahony J. (2018). In Vitro Characteristics of Phages to Guide “Real Life” Phage Therapy Suitability. Viruses.

[5] Rohde C., Resch G., Pirnay J.-P., Blasdel B.G., Debarbieux L., Gelman D., Górski A., Hazan R., Huys I., Kakabadze E. (2018). Expert Opinion on Three Phage Therapy Related Topics: Bacterial Phage Resistance, Phage Training and Prophages in Bacterial Production Strains. Viruses.

[6] Mapes A.C., Trautner B.W., Liao K.S., Ramig R.F. (2016). Development of expanded host range phage active on biofilms of multi-drug resistant *Pseudomonas aeruginosa*. Bacteriophage.

[7] Ross A., Ward S., Hyman P. (2016). More Is Better: Selecting for Broad Host Range Bacteriophages. Front. Microbiol..

[8] Díaz-Muñoz S.L. (2017). Viral coinfection is shaped by host ecology and virus-virus interactions across diverse microbial taxa and environments. Virus Evol..

[9] Scanlan P.D., Buckling A. (2012). Co-evolution with lytic phage selects for the mucoid phenotype of *Pseudomonas fluorescens* SBW25. ISME J..

[10] Scanlan P.D., Hall A.R., Lopez-Pascua L.D.C., Buckling A. (2011). Genetic basis of infectivity evolution in a bacteriophage. Mol. Ecol..

[11] Laanto E., Hoikkala V., Ravantti J., Sundberg L.R. (2017). Long-term genomic coevolution of host-parasite interaction in the natural environment. Nat. Commun..

[12] Kim J.-S. (2018). Microbial warfare against viruses. Science.

[13] Roychoudhury P., Shrestha N., Wiss V.R., Krone S.M. (2013). Fitness benefits of low infectivity in a spatially structured population of bacteriophages. Proc. R. Soc. B Biol. Sci..

[14] De Paepe M., Hutinet G., Son O., Amarir-Bouhram J., Schbath S., Petit M.A. (2014). Temperate Phages Acquire DNA from Defective Prophages by Relaxed Homologous Recombination: The Role of Rad52-Like Recombinases. PLoS Genet..

[15] Barr J.J., Auro R., Furlan M., Whiteson K.L., Erb M.L., Pogliano J., Stotland A., Wolkowicz R., Cutting A.S., Doran K.S. (2013). Bacteriophage on mucus provide immunity. Proc. Natl. Acad. Sci. USA.

[16] Costello E.K., Lauber C.L., Hamady M., Fierer N., Gordon J.I., Knight R. (2009). Bacterial Community Variation in Human Body Habitats Across Space and Time. Science.

[17] Chabas H., van Houte S., Høyland-Kroghsbo N.M., Buckling A., Westra E.R. (2016). Immigration of susceptible hosts triggers the evolution of alternative parasite defence strategies. Proc. R. Soc. B Biol. Sci..

[18] Hall A.R., Scanlan P.D., Morgan A.D., Buckling A. (2011). Host-parasite coevolutionary arms races give way to fluctuating selection. Ecol. Lett..

[19] Scanlan P.D., Buckling A., Hall A.R. (2015). Experimental evolution and bacterial resistance: (Co)evolutionary costs and trade-offs as opportunities in phage therapy research. Bacteriophage.

[20] Torres-Barceló C., Franzon B., Vasse M., Hochberg M.E. (2016). Long-term effects of single and combined introductions of antibiotics and bacteriophages on populations of *Pseudomonas aeruginosa*. Evol. Appl..

[21] Chibeu A., Agius L., Gao A., Sabour P.M., Kropinski A.M., Balamurugan S. (2013). Efficacy of bacteriophage LISTEX^TM^P100 combined with chemical antimicrobials in reducing *Listeria monocytogenes* in cooked turkey and roast beef. Int. J. Food Microbiol..

[22] Allen R.C., Popat R., Diggle S.P., Brown S.P. (2014). Targeting virulence: Can we make evolution-proof drugs?. Nat. Rev. Microbiol..

[23] Seed K.D., Yen M., Jesse Shapiro B., Hilaire I.J., Charles R.C., Teng J.E., Ivers L.C., Boncy J., Harris J.B., Camilli A. (2014). Evolutionary consequences of intra-patient phage predation on microbial populations. eLife.

[24] Laanto E., Bamford J.K.H., Laakso J., Sundberg L.-R. (2012). Phage-driven loss of virulence in a fish pathogenic bacterium. PLoS ONE.

[25] Evans T.J., Trauner A., Komitopoulou E., Salmond G.P.C. (2010). Exploitation of a new flagellatropic phage of *Erwinia* for positive selection of bacterial mutants attenuated in plant virulence: Towards phage therapy. J. Appl. Microbiol..

[26] Betts A., Kaltz O., Hochberg M.E. (2014). Contrasted coevolutionary dynamics between a bacterial pathogen and its bacteriophages. Proc. Natl. Acad. Sci. USA.

[27] Pires D.P., Cleto S., Sillankorva S., Azeredo J., Lu T.K. (2016). Genetically Engineered Phages: A Review of Advances over the Last Decade. Microbiol. Mol. Biol. Rev..

[28] Ojala V., Laitalainen J., Jalasvuori M. (2013). Fight evolution with evolution: Plasmid-dependent phages with a wide host range prevent the spread of antibiotic resistance. Evol. Appl..

[29] Chan B.K., Sistrom M., Wertz J.E., Kortright K.E., Narayan D., Turner P.E. (2016). Phage selection restores antibiotic sensitivity in MDR *Pseudomonas aeruginosa*. Sci. Rep..

[30] Chan B.K., Turner P.E., Kim S., Mojibian H.R., Elefteriades J.A., Narayan D. (2018). Phage treatment of an aortic graft infected with *Pseudomonas aeruginosa*. Evol. Med. Public Heal..

[31] Day T., Read A.F., Shaw M., Hobbelen P., Oliver R., Evans H. (2016). Does High-Dose Antimicrobial Chemotherapy Prevent the Evolution of Resistance?. PLOS Comput. Biol..

[32] Trasta A. (2018). Personalized medicine and proper dosage: Over- and undertreatment of chronic diseases endanger patients’ health and strain public health systems. EMBO Rep..

[33] Pirnay J.P., Verbeken G., Ceyssens P.J., Huys I., de Vos D., Ameloot C., Fauconnier A. (2018). The magistral phage. Viruses.

[34] Dennehy J.J. (2012). What Can Phages Tell Us about Host-Pathogen Coevolution?. Int. J. Evol. Biol..

[35] Gómez P., Bennie J., Gaston K.J., Buckling A. (2015). The Impact of Resource Availability on Bacterial Resistance to Phages in Soil. PLoS ONE.

[36] Meaden S., Paszkiewicz K., Koskella B. (2015). The cost of phage resistance in a plant pathogenic bacterium is context-dependent. Evolution.

[37] Koskella B., Thompson J.N., Preston G.M., Buckling A. (2011). Local biotic environment shapes the spatial scale of bacteriophage adaptation to bacteria. Am. Nat..

[38] Gurney J., Aldakak L., Betts A., Gougat-Barbera C., Poisot T., Kaltz O., Hochberg M.E. (2017). Network structure and local adaptation in co-evolving bacteria-phage interactions. Mol. Ecol..

[39] Henry M., Lavigne R., Debarbieux L. (2013). Predicting in vivo efficacy of therapeutic bacteriophages used to treat pulmonary infections. Antimicrob. Agents Chemother..

[40] De Sordi L., Khanna V., Debarbieux L. (2017). The Gut Microbiota Facilitates Drifts in the Genetic Diversity and Infectivity of Bacterial Viruses. Cell Host Microbe.

[41] Roach D.R., Leung C.Y., Henry M., Morello E., Singh D., Di Santo J.P., Weitz J.S., Debarbieux L. (2017). Synergy between the Host Immune System and Bacteriophage Is Essential for Successful Phage Therapy against an Acute Respiratory Pathogen. Cell Host Microbe.

[42] Sanjuán R. (2017). Collective Infectious Units in Viruses. Trends Microbiol..

[43] Weitz J.S., Poisot T., Meyer J.R., Flores C.O., Valverde S., Sullivan M.B., Hochberg M.E. (2013). Phage-bacteria infection networks. Trends Microbiol..

